# Hepatitis C virus genotypes in Pakistan: a systemic review

**DOI:** 10.1186/1743-422X-8-433

**Published:** 2011-09-08

**Authors:** Sobia Attaullah, Sanaullah Khan, Ijaz Ali

**Affiliations:** 1 Department of Zoology, Islamia College (Public Sector University) Peshawar, Khyber Pakhtunkhwa, Pakistan; 2 Kohat University of Science and Technology, Kohat Khyber Pakhtunkhwa, Pakistan; 3 Institution of Biotechnology and Genetic Engineering, KPK Agricultural University Peshawar, Pakistan

**Keywords:** HCV, Genotypes, Prevalence, Pakistan

## Abstract

**Background and aim:**

Phylogenetic analysis has led to the classification of hepatitis C virus (HCV) into 1-6 major genotypes. HCV genotypes have different biological properties, clinical outcome and response to antiviral treatment and provide important clues for studying the epidemiology, transmission and pathogenesis. This article deepens the current molecular information about the geographical distribution of HCV genotypes and subgenotypes in population of four provinces of Pakistan. 34 published papers (1996-2011) related to prevalence of HCV genotypes/serotypes and subgenotypes in Pakistan were searched.

**Result:**

HCV genotype/s distribution from all 34 studies was observed in 28,400 HCV infected individuals in the following pattern: 1,999 (7.03%) cases of genotype 1; 1,085 (3.81%) cases of genotype 2; 22,429 (78.96%) cases of genotype 3; 453 (1.59%) cases of genotype 4; 29 (0.10%) cases of genotype 5; 37 (0.13%) cases of genotype 6; 1,429 (5.03%) cases of mixed genotypes, and 939 (3.30%) cases of untypeable genotypes. Overall, genotype 3a was the predominant genotype with a rate of 55.10%, followed by genotype 1a, 3b and mixed genotype with a rate of 10.25%, 8.20%, and 5.08%, respectively; and genotypes 4, 5 and 6 were rare. Genotype 3 occurred predominately in all the provinces of Pakistan. Second more frequently genotype was genotype 1 in Punjab province and untypeable genotypes in Sindh, Khyber Pakhtunkhwa and Balochistan provinces.

## Background

Hepatitis C virus (HCV) infection, the sole member of the genus *Hepacivirus*, in the family *Flaviviridae *[1], is a global public health problem [1-3] and is accountable for the second most common cause of viral hepatitis [4]. Pakistani HCV serofrequency figures are significantly higher (4.7%, varying from 0.4%-33.7%) when compared to the corresponding populations in surrounding countries like India (0.66%), Nepal (1.0), Myanmar (2.5%), Iran (0.87%) [5], China (1%) [6] and Afghanistan (1.1%) [7].

HCV has been recognized to be both hepatotropic and lymphotropic virus [8]. The complete HCV genome was determined by Choo *et al *in 1991 and the consensual international classification was made in 1994 [6]. HCV genome is comprised of linear single stranded RNA molecule of positive polarity of ~9.6 kb [1]. The RNA encodes a large polyprotein of about 3,000 amino acids in a single continuous open reading frame (ORF) which is flanked at the 5' and 3' ends by nontranslated regions (5' UTR) [1,2,9]. The ORF comprised of 3 structural genes (C, E1, E2) and 4 nonstructural genes (NS2, NS3, NS4 and NS5) [2,9]. Mutation rate are different in different region of HCV genome. High rate of mutation occur in two region of Envelop E2 glycoprotein, designated hyper variable region 1 and 2 [10]. Of the genome, 5'UTR, the most highly conserved region and thus has been used is most laboratories to develop sensitive detection assays for HCV RNA [2,9]. RNA exhibits a significant genetic, heterogeneity with nucleotide substitution rate of 1.44 × 10^-3 ^and 1.92 × 10^-3 ^per site per year [11].

HCV isolates show four levels of genetic variability: types (65.7% - 68.9% nucleotide sequence identities of full length sequences), subtypes (76.9% - 80.1% nucleotide sequence identities of full length sequences), isolates, and quasispecies (90.8%- 99% nucleotide sequence identities of full length sequences) [6]. Epidemiologically and clinically HCV is classified into 11 genotypes and more than 100 subtypes designated as a, b, c. etc. [1,6,12]. It has been regarded that genotypes 7 through 11 should be consider as variants of the same group and classified as a single genotype, type 6 [13,14].

Genetic heterogeneity is an outstanding feature of HCV and this may have important implications in diagnosis, pathogenesis, treatment, and vaccine development. The determination of HCV genotypes, subtypes and isolates has been helpful in understanding the evolution and the epidemiology of the HCV, and is an important factor in the pretreatment evaluation of patients [8,15,16]. The HCV genotypes show a distinctive geographical distribution [6,9,15]. The global epidemiology of the HCV genotypes is shown in Table 1[13,14].

**Table 1:** Geographic Distribution of HCV Genotypes Worldwide

HCV Genotypes	Geographical distribution
1	United States, Europe, Japan

2	Northern Italy, North America, Europe, and Japan

3	India, Europe, United States

4	North Africa and the Middle East

5	South Africa

6	Hong Kong

7,8,9	Vietnam

10,11	Indonesia

In Pakistan, few studies have been conducted in order to figure out the distribution of various HCV genotypes. This study initiated to find out the molecular epidemiology of various HCV genotypes and subtypes present across the country in all four provinces of Pakistan.

## Literature search

Literature was searched and reviewed in 2011 to summarize scientific reports on distribution, prevalence and epidemiology of HCV genotypes in Pakistan. Articles indexed in the PubMed database and google scholar. Papers published during years 1996 to 2011 were searched by using the following key words: HCV, hepatitis C virus genotypes, epidemiology, prevalence, Pakistan.

A total of 34 original articles on HCV genotype in Pakistan were found; they covered all four provinces. Altogether 28,400 HCV affected individuals were reported in these articles. In this study, 3,887 cases of positive HCV individuals were investigated from Punjab, 2,868 cases from Sindh, 1,200 cases from Khyber Pakhtunkhwa (formerly NWFP) and 152 individuals was available from Balochistan, while 20,417 individuals were investigated from different parts of Pakistan (Table 2).

**Table 2:** Distribution of HCV Genotypes in 34 studies from Pakistan

Ref. No	HCV Genotype frequency (%)							
	
	1	2	3	4	5	6	untypeable	mixed
[17]	1(6.66)	-	13(80)	-	-	-	1(13.33)	-

[18]	4(6.66)	1(2.22)	a = 39(87)	1(2.22)	-	-	-	-

[19]	18(6.4)	3(1.07)	a = 171(61.5)	-	2(0.9)	63(22.6)	-	-

[20]	31(10)	6(1.96)	a = 198(64.7)	1(0.32)	7(2.28)	51(16.6)	6(1.96)	

[21]	20(5.6)	-	241(82.5)	15(4.2)	-	-	64(18)	-

[22]	-	-	314(100)	-	-	-	-	-

[23]	-	-	a = 8(89), b = 1(11)	-	-	-	-	-

[24]	57(7.9)	8(12)	636(87.8)	36(5)	-	6(0.8)	-	-

[15]	a = 10(10.2)	a = 3(3.1)	a = 62(63.3), b = 8(8.2)	-	-	-	11(11.2)	4(4.08)

[16]	b = 21(13.5)	-	a = 80(51.6), b = 43(27.7)	-	-	-	5(3.22)	6(3.87)

[25]	10(31)	-	18(56)	-	-	-	-	4(13)

[26]	8(9.5)	2(2.4)	68(81)	-	-	-	6(7)	

[27]	80(4.84)	154(9.33)	1220(73.85)	41(2.84)	-	-	106(6.42)	51(3.09)

[28]	a = 280(8.3), b = 101(3.0), c = 5(0.15),	a = 252(7.5), c = 3(0.09)	a = 1664(49.05), b = 592(17.66), c = 25(0.15)	50(1.49)	a = 6(0.18)	4(0.12)	201(5.99)	161(4.8)

[29]	a = 10(2.9), b = 5(0.15)	-	a = 242(70.3), b = 19(5.52)	-	3(0.87)	-	52(15.11)	9(2.61)

[30]	a = 2(7.14)	-	a = 14(50), b = 3(10.72)	-	-	-	9(32.14)	-

[31]	b = 10(4.81)	-	a = 107(51.44), b = 33(15.87)	-	-	-	8(3.84)	50(24.04)

[32]	b = 10(4.5)	-	74(33.7)	-	-	-	8(3.6)	19(8.7)

[33]	a = 4(2)	-	a = 115(57.5), b = 6(3)	-	-	-	34(17)	41(20.5)

[2]	a = 1(1.07)	-	a = 81(87.09)	-	-	-	9(9.67)	2(2.14)

[3]	-	-	a = 8(38)	-	-	-	11(52.4)	2(9.6)

[34]	a = 8(9.63), b = 2(2.4)	-	a = 34(40.96), b = 13(15.66)	-	-	-	2(2.4)	24(28.91)

[35]	a = 2(1.5), b = 1(0.8), c = 2(1.5)	a = 1(0.8)	a = 105(81.4), b = 12(9.3), k = 3(2.3)	a = 3(3.2)	-	-	-	-

[1]	a = 12(10), b = 6(5), c = 1(0.8)	a = 8(6.7)	a = 58(48.7), b = 24(20)	-	-	-	3(2.5)	7(5.9)

[36]	a = 678(3.3), b = 170(0.83), c = 49(0.24)	a = 431(2.1), b = 48(0.23), c = 3(0.01)	a = 12537(74.2), b = 18834(10.9), c = 50(0.24)	101(0.49	a = 13(0.07)	a = 12(0.06)	-	965(4.7

[13]	a = 9(2.17), b = 3(0.722)	-	a = 234(56.38), b = 25(6.02)	-	-	-	116(27.95)	28(6.74)

[37]	-	a = 1(2.1)	a = 28(65.1), b = 4(9.3)	-	a = 5(11.6)	a = 5(11.6)	-	-

[6]	a = 322(23.6)	-	a = 763(55.9), b = 43(3.2)	a = 170(12.5), b = 16(1.17)	-	-	34(2.5)	16(1.2)

[38]	a = 1(4), b = 2(8)	a = 4(16), b = 1(4)	a = 11(44), b = 3(12)	-	-	-	3(12)	-

[4]	a = 3(1.5), b = 5(2.5)	a = 78(39), b = 2(1)	a = 62(31), b = 16(8)	-	-	-	34(17)	-

[39]	a = 5(7.9)	a = 22(34.9)	a = 18(28.6)	9(14.3)	-	-	9(14.3)	-

[14]	a = 10(5.4)	a = 15(8.1)	a = 63(34.1), b = 13(7)	-	-	-	70(37.8)	14(7.6)

[40]	a = 1(4.34)	-	a = 17(73.91), b = 3(13.04)	-	-	-	1(4.34)	1(4.34)

[41]	a = 3(13)	-	a = 15(65), b = 4(17)	-	-	-	1(4)	-

## Discussion and Conclusion

HCV has come to the top of virus-induced liver diseases in many parts of the world and has gained endemic proportions in our population but there is no national data collection system for evaluation of genotypes of HCV infection. The data on geographic distribution of genotypes of HCV is among largest of its kind from Pakistan. This is perhaps the first study done in area of geographic distribution of genotypes of HCV in all four provinces of Pakistan. Pakistan is situated in the western part of the Indian subcontinent, with Afghanistan and Iran on the west, India on the east, and the Arabian Sea on the south. This geographical situation, mass immigration and illegal drug traffic from Afghanistan, absence of good preventive measures in the past e.g. extensive reuse of non-sterilized syringes, fragile health structure, unscreened blood transfusion, use of contaminated razor by barber, general poverty and poor education have all affected epidemiology of HCV in our country. Pakistan consists of four provinces; Balochistan is geographically largest of four provinces but has smallest population; Punjab is second largest province and has largest population, Sindh is third largest province geographically and KPK is geographically the smallest of four provinces, which is bordered on to the west by Afghanistan. The immigration of populations from countries of high endemicity of the infection seems to contribute significantly to the rapid modification of epidemiological data, especially in areas bearing the high burden of immigrants.

**Figure 1:**
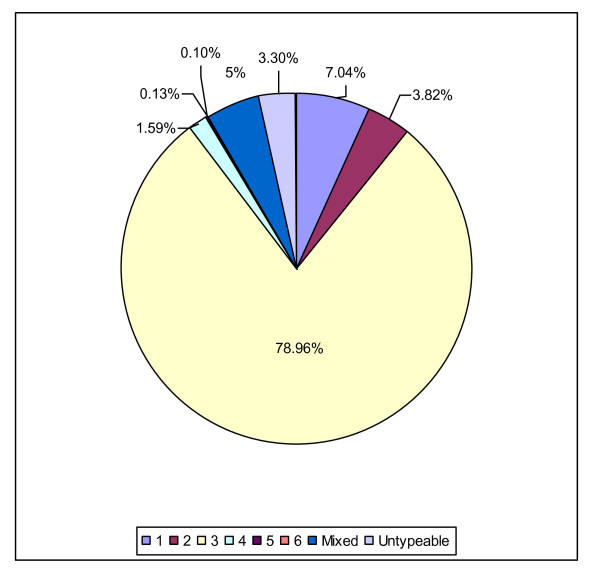
**Distribution of HCV Genotypes in Pakistan**.

**Figure 2:**
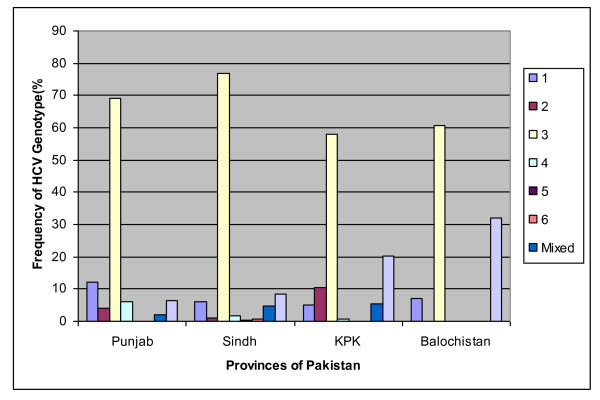
**Distribution of HCV Genotypes in all provinces of Pakistan**.

Genotype 1 was accounted 7.03% of the cases and subtype 1a predominated with a rate of 4.82%. Genotype 2 had frequency of 3.81% and most frequent subtype was 2a (2.89%). Genotype 3 alone contributed to at least 78.96% of all isolates and most frequent subtype was 3a (58.01%), followed by 3b (9.76%). Genotype 4, 5 and 6 were rare with the rate of 1.59%, 0.10% and 0.13, respectively. The rates for mixed genotype, and untypeable genotypes were 5.03%, and 3.30%, respectively (Table 3, Figure 1 and 2 showed distribution of genotypes/subtypes in Pakistan). HCV genotypes of mixed infection have been investigated in only 118 (8.25%) individuals and mixed subtypes in 157 (10.98%) individuals. Mixed infection 1&3 was found with the rate of 0.35%, 1&4 (0.003%), 3&6 (0.021%), 2&3 (0.017%), 3&5 (0.007%), 3&4 (0.003%), 1&5 (0.007%) and 2&4&6 (0.007%). The most frequent mixed subtype was 3a&3b (0.387%), followed by 1a&3a (0.06%), 1b&3a (0.05), 1a&3b (0.024%), 2b&3a (0.014%) and 2a&3a (0.010%).

**Table 3:** HCV Genotype/subtypes Distribution in Pakistan

Genotype	Frequency	Percentage
1 (all subtypes)	1999	7.038

1(Undefined subtypes)	237	0.834

1a	1369	4.82

1b	336	1.183

1c	57	0.2

2 (all subtypes)	1085	3.818

2 (Undefined subtypes)	180	0.633

2a	821	2.89

2b	78	0.274

2c	6	0.021

3 (all subtypes)	22429	78.964

3 (Undefined subtypes)	3104	10.92

3a	16475	58.01

3b	2772	9.76

3c	75	0.264

3k	3	0.01

4 (all subtypes)	453	1.594

4 (Undefined subtypes)	264	0.929

4a	173	0.609

4b	16	0.056

5 (all subtypes)	29	0.101

5 (Undefined subtypes)	5	0.017

5a	24	0.084

6(all subtypes)	37	0.13

6 (Undefined subtypes)	25	0.088

6	12	0.042

Undefined subtypes	939	3.306

Mixed	1429	5.031

Total	28400	100

This study showed that HCV infections in hepatitis patients are attributed predominantly to viral genotype 3 in Pakistan with the rate of 68.94% in Punjab, 76.88% in Sindh, 58% in KPK and 60.71% in Balochistan. In addition, there was a relatively high prevalence of genotype 1 (12.14%) in Punjab while untypeable infection represented the second more frequent genotype among the studied population in Sindh (8.33%), KPK (20.16%) and Balochistan (32.14%).

Statistically no difference was found in HCV genotypes in terms of age and sex of the patients, also confirmed by other studies [14,42,43] but in contrast to reports from developed countries like USA and Southeast Asia, where lifestyles among young adults seem to have influenced the molecular epidemiology of HCV especially by their young drug addicts [42].

With reference to this countrywide information this study suggested that HCV 3 was the leading genotype in Pakistan. Other major types were genotype 1 and mixed genotype. A high prevalence of variants of genotype 3 alone to be over 79.43% in Pakistan overall- alone or in combination with another genotype (78.96% had genotype alone, and a further 157/157 had this genotype mixed with another genotype). High prevalence of HCV genotype 3 in Pakistan is a good hope for cure as well as control of HCV infection [6,15]. Genotype 3 requires shorter duration of treatment as compared with genotype 1, with its associated reduced cost and side effects [33]. The predominance of HCV genotype 3 in our population confirmed the predominance of HCV genotype 3 in the surrounding countries including India [2,15,16], Iran [44], Bangladesh [14,15] and China [2].

Each genotype has few subtypes in Pakistan, three for Genotype 1, three for genotype 2, four for genotype 3, two for genotype 4, one for genotype 5 and one for genotype 6. This showed reduced diversity of subtypes in Pakistan. It would therefore appear from different studies conducted in different parts that substantial regional differences do exist in our country. Genotype 1 is the second highest genotype in the country. Balochistan shares a long border with Iran in the west where genotypes 1a and 3a are most prevalent [3,45]. High prevalence of genotype 3a and 1b has been reported from China [2]. It is quite possible that genotype 1 may have entered into Pakistan from these countries through local persons who cross borders for job and trade. Shifts in HCV genotype distribution needs to be paid more attention. This raises an alarming signal on the major steps to be taken to reduce such infection as genotype 1a and 4a are associated with severe cirrhosis.

A proportion of patients will have their genotype reported untypeable (3.306%). Presence of untypeable samples in indicated that some novel genotypes are present in Pakistan. Our next goal should be to find out the sequencing of these samples, which will be very useful in prescribing the therapy for patients [1]. For being able to identify untypeable genotypes, phylogenetic analysis of extensive genome sequences is required and continuous efforts are required toward a better characterization of this variant. Most of the studies were not able to separate different kinds of patients among the untypeable genotype group because of the retrospective nature of their studies. Other studies were unable to sequence the untypeable samples due to lack of sequencing facility.

## Competing interests

The authors declare that they have no competing interests.

## Authors' contributions

SA and SK designed, analyzed and draft the manuscript. IA critically studied the manuscript. All authors read and approved the final manuscript.
